# Impact of a maternal health voucher scheme on institutional delivery among low income women in Pakistan

**DOI:** 10.1186/1742-4755-8-10

**Published:** 2011-05-03

**Authors:** Sohail Agha

**Affiliations:** 1 Population Services International, 1120 19th Street, NW, Suite 600, Washington DC 20036, USA

## Abstract

**Background:**

Only 39% of deliveries in Pakistan are attended by skilled birth attendants, while Pakistan's target for skilled birth attendance by 2015 is > 90%.

**Methods:**

A 12-month maternal health voucher intervention was implemented in Dera Ghazi Khan City, located in Southern Punjab, Pakistan in 2009. A pre-test/post-test non-experimental study was conducted to assess the impact of the intervention. Household interviews were conducted with randomly selected women who delivered in 2008 (the year prior to the voucher intervention), and with randomly selected women who delivered in 2009. A strong outreach model was used and voucher booklets valued at $50, containing redeemable coupons for three antenatal care (ANC) visits, a postnatal care (PNC) visit and institutional delivery, were sold for $1.25 to low-income women targeted by project workers. Regression analysis was conducted to determine the impact of the voucher scheme on ANC, PNC, and institutional delivery. Marginal effects estimated from logistic regression analyses were used to assess the magnitude of the impact of the intervention.

**Results:**

The women targeted by voucher outreach workers were poorer, less educated, and at higher parity. After adjusting for these differences, women who delivered in 2009 and were sold voucher booklets were significantly more likely than women who delivered in 2008 to make at least three ANC visits, deliver in a health facility, and make a postnatal visit. Purchase of a voucher booklet was associated with a 22 percentage point increase in ANC use, a 22 percentage point increase in institutional delivery, and a 35 percentage point increase in PNC use.

**Conclusions:**

A voucher intervention implemented for 12 months was associated with a substantial increase in institutional delivery. A substantial scale-up of maternal health vouchers that focus on institutional delivery is likely to bring Pakistan closer to achieving its 2015 target for institutional delivery.

## Background

The pace of decline of the Maternal Mortality Ratio (MMR) in Pakistan has been slower than for the rest of South Asia. India, which has experienced a rapid increase in skilled birth attendance in recent years, is driving the decline in the region [1]. New approaches to improving maternal health service delivery are needed in Pakistan, if the country is to meet the Millennium Development Goal (MDG) target of reducing the MMR by 75% of its 1990 level, or to 140 maternal deaths per 100,000 live births.

With many developing countries failing to achieve the level of progress needed to meet their MDG targets, international donors are interested increasingly in linking the financing of health services to the achievement of results, rather than to inputs. Performance-based Financing (PBF), also referred to as Pay for Performance (P4P) or Results-based Financing (RBF), is the use of financing to encourage the achievement of agreed-upon results. An often cited example of PBF is of a provider being paid for outcomes such as an increase in immunization coverage rather than inputs like the number of immunization syringes [2,3].

PBF has been used widely in the United States and United Kingdom to drive quality improvements in health care service delivery. Other health systems in developed countries are also in the process of adopting this approach [4]. In developing countries, an important focus of PBF schemes is the increased use of health services by vulnerable groups in order to meet MDG targets, and to increase the accountability, efficiency and quality of services [3].

To date, there has been limited research on the effectiveness of PBF schemes in both high and low-income countries [5]. When research has been conducted, it has shown mixed results [4,6] or the research design has not been strong enough to separate the effect of PBF from other, simultaneous changes occurring in the health system at the same time [7]. The problem of the lack of an evidence base on PBF initiatives is particularly acute in low-income countries [3]. Reviewers have highlighted the need for systematic research to determine the effectiveness of PBF approaches in less developed health systems.

The present study assesses the impact of a PBF project to increase the utilization of maternal health services in a small city in Pakistan. Across Pakistan, about 44% of women living in small urban areas have their babies delivered by skilled birth attendants, [8]. This is far below the Pakistan government's target of greater than 90% of deliveries being attended by skilled birth attendants by 2015. Socio-economic differentials in the use of skilled birth attendants are large and persistent in Pakistan. A recent study that assessed the impact of well-funded maternal health project in 10 districts of Pakistan found no change in institutional delivery among women in the poorest economic group [9]. Since maternal deaths tend to clustered among the poor in developing countries [10], substantial reductions in maternal mortality are unlikely to occur in the absence of increases in skilled birth attendance among the poorest women.

### Performance-based financing (PBF)

PBF is defined as the transfer of money or material goods conditional on taking a measurable action. It includes a wide range of interventions that vary in terms of the level at which the incentives are targeted (e.g. health facility, individual providers, recipients of health care), the targeted results (e.g. improved quality of care, delivery at a health facility), indicators used to measure results (e.g. service statistics, population level outcomes), choice of targets (e.g. payment per immunization, payment by achievement of a certain level of immunization coverage in a population), magnitude of the incentive (e.g. partial subsidy for delivery at a facility, complete subsidy for delivery and reimbursement of transport cost), and type of the incentive (e.g. cash payments, material goods, free services) [11]. PBF approaches may target either the demand or the supply-side of health service provision [7]. In the case of demand-side financing, government or donor money goes directly to low-income consumers in the form of a subsidy that enables them to purchase services. In the case of the supply-side financing, government or donor subsidy goes to the provision of services, traditionally in the public sector [12]. Supply-side financing approaches are the standard approach used in the provision of maternal care by the public sector in many developing countries, including Pakistan, where providers receive funds for ensuring access to care, either free of charge or at highly subsidized rates [13].

### Vouchers

Vouchers are a specific demand-side financing mechanism that can be used to target essential health services to vulnerable populations such as poor, pregnant women and to protect them from catastrophic expenditures such as emergency obstetric care [13]. In a voucher scheme, the consumer receives a booklet or token that covers all or part of the price of a package of services [12]. Because they are highly targeted, voucher interventions are expected to improve health outcomes among the poor.

### Development of the voucher intervention

#### The context

The Pakistan Demographic and Health Survey conducted in 2006-07 (PDHS 2006-07) showed that despite an increase in antenatal care (ANC) visits during the last two decades, only one-third of women in cities outside major urban centers (hereafter referred to as "small urban areas") made the four antenatal visits recommended by WHO [8].

In small urban areas, only half of all pregnant women prepared for childbirth by discussing with their husbands the place of delivery or by setting aside money in the case of an emergency. And only 40% delivered in a health facility. The consequences of delivering outside a health facility were potentially quite severe for these women: 74% of women in small urban areas who delivered at home reported that an unboiled thread was used to tie the cord; 40% reported the use of a pair of scissors, knives, or old razor blades to cut the umbilical cord [8].

#### Obstacles to the utilization of maternal health services

A range of factors contribute to the low levels of utilization of maternal health services in small urban areas of Pakistan. The PDHS 2006-07 showed that awareness of the importance of ANC was low: more than three out of four women who did not make an ANC visit thought it unnecessary; and one out of four women reported the cost of ANC being prohibitive [8].

The two most common reasons cited by women for not delivering in a health facility were the lack of perceived benefit of doing so (65%) and the prohibitive cost (29%).

Nearly half of women in small urban areas reported that *dais *(traditional birth attendants lacking formal health training) assisted them in home deliveries [8]. *Dais *are informal providers who are members of the community and are easily accessible to poor women. In some cases, they may have delivered babies for a family for several generations. Home deliveries are consistent with conservative values in many parts of Pakistan that emphasize the importance of *purdah *(the practice of preventing women from being seen by men who are not immediate kin). Because *dais *are local women from a similar social background, low-income women find it easier to communicate with them than with medical practitioners.

#### Using vouchers to address the barriers to utilization of maternal health services

Planners of the voucher project felt that a demand-side strategy that removed social and cultural barriers associated with obtaining care from a medical facility, and also lowered the monetary cost associated with utilizing these services would likely succeed in increasing the use of maternal health services [14]. To achieve this, a behavior change strategy to increase awareness of the benefits of ANC, institutional delivery, and postnatal care (PNC) combined with a PBF mechanism allowing low-income women to overcome financial obstacles to seeking care was implemented.

### The setting: Dera Ghazi Khan City

Located in southern Punjab, Dera Ghazi Khan (D.G. Khan) is one of the poorest districts in Pakistan with a poverty incidence of 70% [15]. In 2008, it had an estimated population of 2.2 million. Approximately, 15% of the population of D.G. Khan district is urban and is located in two towns: D.G. Khan City and Taunsa Sharif. A representative household survey of D.G. Khan district conducted in 2005 showed that 57% of pregnant women living in the two cities of the district delivered at a health facility. About 48% of women in urban D.G. Khan had made three or more ANC visits during their last pregnancy, and 25% had made a PNC visit [16]. In October 2008, a PBF intervention of 12-month duration was launched in D. G. Khan City with funds from the USAID-supported PAIMAN project. The objectives of the project were to increase the use of ANC, PNC and institutional delivery.

### The intervention

The scope of the pilot project was to provide a package of maternal health services to 2,000 pregnant low-income women in D.G. Khan City. This city had an estimated population of 258,000 in 2008. Based on the crude birth rate of 30 per 1,000 population for small urban areas of Pakistan [8] about 7,700 births were expected in D.G. Khan city in 2008. Hence, if all 2,000 women who participated in the voucher scheme were to use their coupon for institutional delivery, 26% coverage could be expected. The package of services available to study participants included three ANC visits, normal delivery or referral for caesarian-section, and a PNC visit. In addition, clients could get their complete blood count and an ultrasound examination.

The services were available during the 12-month period, beginning October 1 2008 through September 30, 2009 from private providers who were part of a network managed by the NGO, Greenstar Social Marketing. Under the Goodlife brand, Greenstar has a national network of 7,500 private providers who have been trained in the provision of ANC, PNC, emergency obstetric care, neonatal care, child care, and family planning services. Quality assurance visits to network providers are made by the Greenstar medical team on a quarterly basis. Based on an assessment of their capacity to provide quality maternal and child health services, 22 Goodlife providers located in D.G. Khan City were preapproved for provision of services under this scheme.

No cash payments were made by voucher recipients to providers. Instead, voucher recipients made a one-time payment of US $1.25 to Greenstar outreach workers who sold them the voucher booklet. The voucher booklet contained coupons for services that clients were entitled to receive upon purchase of the booklet. After providing a particular service, the provider would tear off the relevant coupon and submit it to Greenstar for reimbursement. Greenstar would reimburse providers at an agreed-upon rate for individual services within 35 days of submission of a coupon for a particular service. Providers were reimbursed US $1.25 for each ANC and PNC visit, US $31 for a normal delivery, and US $125 for a caesarian delivery. After the approval of individual claims, the Greenstar finance department transferred funds directly to provider bank accounts.

Clients were reimbursed by providers a standard amount for the cost of transportation to the facility. For most types of visits the reimbursement was US 62 cents. For a delivery, the transport reimbursement was US $3. Providers, in turn, were reimbursed by Greenstar for transportation costs at the same time as payments for relevant services were made. Ten percent of coupons were validated by outreach workers who checked with voucher recipients to ensure that they had indeed utilized services. Clients were asked about the care received from providers during this validation. Random checks of voucher coupons were also conducted by the project supervisor.

Voucher recipients were identified through door-to-door visits by project outreach workers. Three criteria were used for respondent selection: physical appearance of neighborhood where a potential recipient's house was located (including the lack of presence of basic amenities such as sanitation), household income below the national poverty line, and no prior experience of delivery in a health facility. The third criterion was developed in order for the project to have optimal public health impact. Vouchers could only be sold to women who met selection criteria established by the project.

After potential recipients had been identified, local elected officials serving on the union council were consulted for verification of recipients' eligibility. Data collected at the time of voucher sales showed that median monthly household income was US $45. Less than 1% of voucher recipients reported a monthly income greater than the government's poverty line (US $75). Ninety-seven percent of voucher recipients who had had at least one birth reported that a *dai *had delivered their last child. Sixty-five percent of voucher recipients reported that their husbands were daily wage laborers.

## Methods

### Study design

A pre-test/post-test non-experimental design was used to assess the pilot. The assessment aimed to compare women who gave birth in the period during which the voucher scheme was implemented to women who gave birth in the period just prior to the intervention. The study was designed to compare the use of ANC, delivery at a health facility, and the use of PNC between these two groups of women.

In all seven union councils in D.G. Khan city, household survey data was collected from a random sample of mothers who had delivered prior to the PBF intervention and from a random sample of mothers who had delivered during the intervention period. A union council is the smallest administrative unit in Pakistan with a population usually varying between 20,000 and 30,000. Informed consent was obtained from female respondents prior to interviewing them, consistent with IRB procedures approved by Tulane University Medical Center IRB.

The household survey was conducted over an 18-day period, from March 27, 2010 to April 13, 2010. From each union council of D.G. Khan city, 100 mothers who had delivered in the period January 2008 to August 2008 (pre-intervention period) and 100 mothers who had delivered in the period January 2009 to August 2009 (intervention period) were randomly selected.

Within each union council, multiple random starting points were chosen, and households listed prior to the selection of eligible respondents. At the household level, a listing was done of married women 15-49 years who had children 36 months or younger. After the woman's name and age were listed, the name and age of her youngest child (in case the woman had more than one child born in the last three years) was determined. A calendar method was used to determine the age of the youngest child. Women were first asked about the year in which their youngest child was born. They were then asked about the month of the year in which their youngest child was born. A woman who gave birth between January 2008 and August 2008 was eligible to be sampled for the study and represented women who had delivered prior to implementation of the voucher scheme. A woman who gave birth between January 2009 and August 2009 was also eligible to be sampled for the study and represented women who delivered during the voucher scheme implementation period.

The above methodology was pre-tested in February 2010 in Taunsa Sharif, another city in D.G. Khan district, with a sample of 30 women. A child's year and month of birth obtained using the calendar method was compared to information obtained from their birth certificates or immunization cards. The comparison showed a high level of comparability between children's ages obtained from the calendar method and children's ages from birth or immunization records.

The final sample consisted of 681 mothers who delivered during 2008 and 741 mothers who delivered during 2009. There was little variation in the population sizes of the seven union councils (populations ranged from 25,999 to 27,928). Accordingly, no weights were attached to the data. The data were collected by AcNielsen Pakistan (Pvt.) Ltd. who have been conducting household surveys in Pakistan since 1991.

### Measures

#### Dependent variables

Three dependent variables were used for this analysis -ANC, institutional delivery and PNC. For ANC, women were coded '1' if they made at least three ANC visits, and '0' if they made fewer visits. For institutional delivery, women were coded as '1' if they delivered at a health facility and coded '0' if they delivered at home. For PNC, women were coded '1' if they made a PNC visit, and '0' if they did not.

#### Participation in the voucher scheme

To identify women who participated in the voucher scheme, respondents to the survey were asked if a health worker had visited their house to tell them about a voucher scheme for pregnant women. Respondents who answered in the affirmative were asked if they had purchased a voucher booklet from the health worker. Purchase of the voucher booklet was used as a measure of participation in the voucher scheme.

#### Independent variables

All independent variables included in the analysis are supported by prior literature on the determinants of ANC, institutional delivery and PNC use. Variables included in the analysis of ANC, institutional delivery, and PNC are mother's age (categorized as less than 24 years, 25-29 years, 30-34 years and greater than 34 years), parity (number of living children), mother's education (none, any primary, middle, secondary, matriculate or higher), mother's autonomy (low, middle, high), wealth quintiles, travel time to the nearest health facility (within 5 minutes), and exposure to mass media (daily television viewership). A recent analysis found the above variables to be important determinants of the use of maternal health services in Pakistan [17].

Wealth quintiles were created in a manner similar to their creation for the Demographic and Health Surveys [18]. Binary variables were created for the following household possessions and amenities: television, tape recorder, washing machine, refrigerator, bicycle, motorcycle, cell phone, computer, water supply, flush connection to sewerage, use of natural gas for fuel. Factor analysis was used to create a wealth factor score, which was divided into quintiles.

The variable measuring mother's autonomy was also created using factor analysis. First, mothers were coded '1' if decisions regarding the following were made by her alone or by the couple together and '0' otherwise: small household expenditures (e.g. toothpaste, batteries, etc.); large household expenditures (e.g. television, refrigerator, etc.); expenditures on women's clothes and jewelry; woman's employment outside the home; purchase or sale of property; children's clothes; where to take children in the case of illness; where to take the mother in case of illness; purchase of medicine; children's education; use of contraception; and visits to relatives. Factor analysis was used to create a female autonomy score, which was divided into terciles. The approach is similar to the approach used to create wealth quintiles.

### Statistical analysis

#### Model development

A multistage process was used to create a base model for the three dependent variables: the use of ANC; delivery at a health facility; and the use of PNC. Bivariate relationships between each independent variable and outcome were investigated using a binary logistic regression model. Those independent variables found to be significant at the bivariate level were included in a multivariate regression model. Each independent variable was tested using an improvement chi-square test to determine if the independent variable improved the fit of the model. If an independent variable did not improve the fit of the model, it was dropped. Thus, the most parsimonious model was built for each outcome variable. In order to make the models comparable, however, any variable that remained in the final model for any of the three dependent variables was retained in all models.

#### Impact analysis

To determine the impact of the voucher scheme on the three outcomes, the 2008 and 2009 data were pooled. A four category variable was created to indicate 1) women who delivered in 2008 (the pre-intervention year), 2) women who delivered in 2009 (the intervention year) but were not contacted by outreach workers, 3) women who delivered in 2009, were contacted by outreach workers but not sold vouchers and 4) women who delivered in 2009 and were sold voucher booklets by outreach workers. The independent effects of purchase of the voucher booklet were estimated after controlling for demographic and socio-economic differences between women who were exposed to the voucher intervention and women who were not. The multi-stage design of the survey was taken into account in the statistical analysis. STATA 10 was used for the statistical analysis. Marginal effects were obtained using a postestimation command in STATA after running the logistic regression models [19]. All marginal effects shown are based on statistically significant odds ratios.

## Results

### Characteristics of women sampled in 2008 and 2009

Table 1 shows the characteristics of mothers who gave birth during 2008 and 2009 in D.G. Khan city. There were no significant differences by age, parity or education. In both 2008 and 2009, the average age of women who gave birth was 27 years. Slightly more than one-fifth of women in each sample delivered their first child and another one-fifth had a fifth or higher order birth. Approximately one-third of women had no education and about one-fifth had matriculate or higher education.

**Table 1:** Characteristics of women who gave birth in D. G. Khan City, pre and post-intervention

	Pre-intervention (2008) % (n = 681) (1)	Post-intervention (2009) % (n = 742) (2)	p-value for chi-square test
**Maternal Factors**			
Mean age of the mother	27.0	26.6	NS
Parity/Living children			
1	21.0	23.0	NS
2	20.6	22.1	
3	15.6	18.7	
4	17.0	14.6	
5 or more	25.8	21.6	
Mother's education			
None	34.7	31.1	NS
Any primary	17.3	15.8	
Middle	14.8	14.4	
Secondary	13.2	18.3	
Matriculate or higher	20.0	20.4	
			
**Household Factors**			
Mother's autonomy			
Low	32.5	33.0	NS
Medium	33.9	31.4	
High	33.6	35.6	
Wealth quintiles			
Fifth/poorest	19.4	19.4	NS
Fourth	19.8	20.4	
Third	20.1	18.9	
Second	18.8	18.6	
First/least poor	21.9	22.8	
			
**Program Factors Unrelated to the Scheme**			
Travel time to nearest health facility			
More than five minutes	66.7	66.4	NS
Five minutes or less	33.3	33.6	
Mass Media Exposure			
Do not watch television daily	33.3	34.2	NS
Watch television daily	66.7	65.8	
			
**Exposure to the voucher scheme**			
No exposure	-	77.2	
Visited by voucher outreach worker	-	9.3	
Visited by worker and purchased voucher	-	13.5	

Total	100.0	100.0	

NS = not significant

There were no differences between the two samples in terms of household factors such as mother's autonomy and household wealth.

There were also no differences between the two samples in terms of programmatic factors unrelated to the voucher scheme: one-third of women lived within five minutes of the nearest health facility; two-thirds of the sample watched television daily.

In 2009, 77% of women who gave birth in DG Khan City were not exposed to the voucher scheme. About 9% of women were visited by an outreach worker but were not sold vouchers. About 13% of pregnant women met the project's eligibility criteria and were sold voucher booklets from outreach workers.

### Characteristics of women who were sold voucher booklets

In Table 2, characteristics of women who were sold a voucher booklet in 2009 are compared to characteristics of women who were contacted by outreach workers but not sold a booklet and to women who were not contacted by outreach workers.

**Table 2:** Characteristics of women who gave birth in 2009, by exposure to the voucher intervention

	Women not contacted by workers (no exposure) (n = 573) (1)	Women not sold voucher booklet (n = 69) (2)	Women sold voucher booklet (n = 100) (3)	p-value for chi-square test
**Maternal Factors**				
Mother's Age				
< 24	35.6	26.1	28.0	NS
25-29	35.1	47.8	33.0	
30-34	19.4	17.4	29.0	
> 34	9.9	8.7	10.0	
Parity/Living children				
1	24.6	23.2	14.0	< 0.01
2	22.5	21.7	20.0	
3	19.5	20.3	13.0	
4	14.8	11.6	15.0	
5 or more	18.5	23.2	38.0	
Mother's Education				
None	30.9	29.0	34.0	< 0.01
Any primary	13.3	18.8	28.0	
Middle	15.2	8.7	14.0	
Secondary	19.0	17.4	15.0	
Matriculate or higher	21.6	26.1	9.0	
				
**Household Factors**				
Mother's Autonomy				
Low	30.2	30.4	30.0	NS
Medium	43.8	55.1	48.0	
High	26.0	14.5	22.0	
Wealth quintiles				
Fifth/poorest	17.3	21.7	30.0	< 0.05
Fourth	20.1	17.4	24.0	
Third	19.0	14.5	21.0	
Second	19.0	18.8	16.0	
First/least poor	24.6	27.5	9.0	
				
**Program Factors Unrelated to the Scheme**				
Travel time to nearest health facility				
More than five minutes	66.0	65.2	70.0	NS
Five minutes or less	34.0	34.8	30.0	
Mass Media Exposure				
Do not watch television daily	33.7	31.9	39.0	NS
Watch television daily	66.3	68.1	61.0	

Total	100.0	100.0	100.0	

NS = not significant

Outreach workers sold voucher booklets to women at higher parity: 38% of women who were sold voucher booklets had five or more children, compared to 23% of women who were not sold a booklet, and 18% of women who were not contacted by outreach workers. Women who were sold a voucher booklet were also less educated and poorer than other women: only 9% of women sold voucher booklets had matriculate or higher education, compared to 26% of women who were not sold a booklet and 22% of women who were not contacted; only 9% of women who were sold voucher booklets were in the first/least poor quintile compared to 27% of women who were not sold booklets and 25% who were not contacted.

Women who were sold voucher booklets did not significantly differ with other women in terms of their age or their level of autonomy. There were also no significant differences between women who were sold booklets and other women in terms of programmatic factors unrelated to the voucher intervention such as travel time to the nearest facility and daily mass media exposure.

### Changes in use of maternal health services by household wealth

Table 3 shows changes in the use of ANC, institutional delivery, and PNC by household wealth quintiles. Columns 1 and 2 in Table 3 show the use of maternal health services by wealth quintiles among women who gave birth in 2008 and among women who gave birth in 2009, respectively. Between 2008 and 2009, use of ANC (i.e. 3 or more ANC visits) increased significantly in all quintiles except the wealthiest quintile. The largest increase (20 percentage points) in ANC use occurred among women in the third quintile, from 61% to 81%. The use of ANC by women in the fifth/poorest quintile increased from 34% to 51%, or by 16 percentage points. There was no significant change in ANC use among women in the first/least poor quintile, which had reached 86% by 2008.

**Table 3:** Changes in ANC, institutional delivery, and PNC use, by household wealth

	Pre-intervention (2008) % (n = 681) (1)	Post-intervention (2009) % (n = 742) (2)	p-value for chi-square test (3)
**ANC (3+ visits)**			
Wealth quintiles			
Fifth/poorest	34.1	51.4	< 0.01
Fourth	50.4	62.9	< 0.05
Third	61.3	81.4	< 0.001
Second	72.7	87.7	< 0.01
First/least poor	85.9	91.1	NS
			
**Institutional Delivery**			
Wealth quintiles			
Fifth/poorest	31.8	53.5	< 0.001
Fourth	48.1	47.0	NS
Third	63.5	70.7	NS
Second	73.4	76.1	NS
First/least poor	84.6	87.0	NS
			
**PNC**			
Wealth quintiles			
Fifth/poorest	12.1	22.9	< 0.05
Fourth	17.0	27.8	< 0.05
Third	33.6	40.0	NS
Second	38.3	46.4	NS
First/least poor	46.3	58.6	< 0.05

NS = not significant

By contrast to changes in ANC use, the increase in the use of a health facility for delivery occurred only among women in the fifth/poorest quintile: institutional delivery increased from 32% to 53% among women in the fifth quintile, or by 21 percentage points. There were no significant changes in institutional delivery among women in any of the other wealth quintiles.

Changes in PNC use occurred among the poorest women as well as among the least poor women: PNC use increased by 11 percentage points among women in the fifth and fourth quintiles, and by 12 percentage points among women in the first quintile.

### Unadjusted effects of exposure to the voucher intervention

#### ANC Use

**Figure 1:**
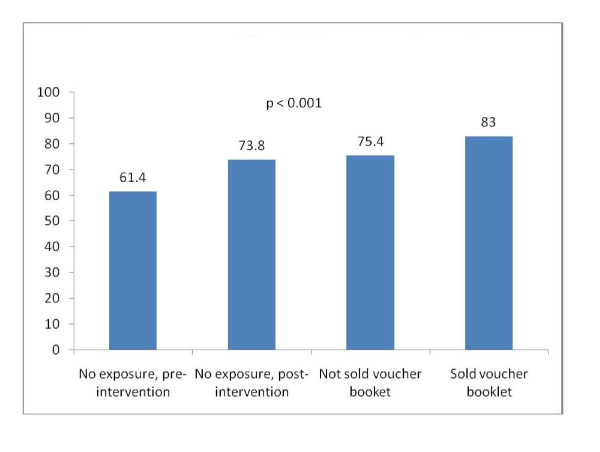
**Use of ANC (3+ visits), by exposure to voucher intervention (n = 1,423)**.

Figure 1 shows the bivariate relationship between exposure to the voucher scheme and use of ANC. Sixty one percent of women who gave birth in 2008 made at least three ANC visits. ANC use was higher among women who gave birth in 2009: ANC use reached 74% among women who were not contacted by voucher outreach workers, 75% among women who were contacted by outreach workers but not sold voucher booklets, and 83% among women who were sold voucher booklets.

#### Institutional Delivery

**Figure 2:**
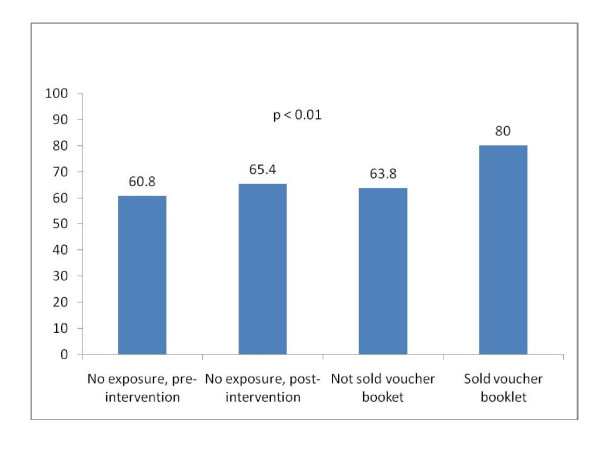
**Delivery in a health facility, by exposure to voucher intervention (n = 1,423)**.

Figure 2 shows the bivariate relationship between exposure to the intervention and delivery at a health facility. Institutional delivery was 61% among women who gave birth in 2008. In 2009, the institutional delivery rate was 65% among those not exposed to the intervention, 64% among women who were not sold voucher booklets, and 80% among women who were sold voucher booklets.

#### PNC Use

**Figure 3:**
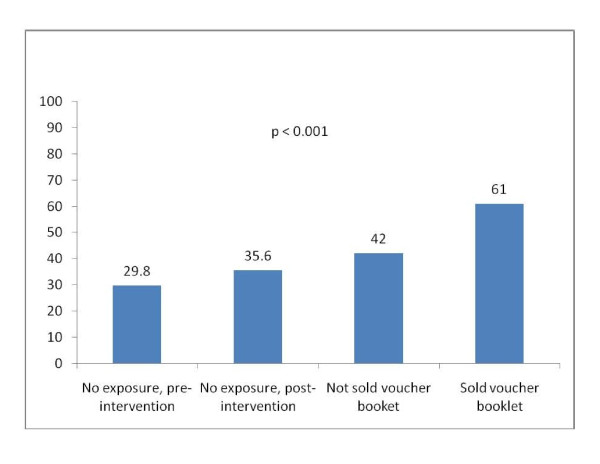
**Use of postnatal care, by exposure to voucher intervention (n = 1,423)**.

Figure 3 shows use of PNC by exposure to the intervention. PNC use was 30% among women who gave birth in 2008. In 2009, it rose to 36% among women not exposed to the intervention, 42% among women contacted by outreach workers but not sold voucher booklets, and 61% among women who were sold voucher booklets.

### Effects of voucher scheme on ANC use

Table 4 shows the impact of the voucher scheme on ANC use. Column 1 of Table 4 shows unadjusted odds ratios associated with a woman having made at least three ANC visits during her last pregnancy. Women who gave birth in 2009 but were not exposed to the voucher intervention had a 1.77 times higher odds of making at least three ANC visits relative to women who gave birth in 2008. This suggests that there was a secular trend in ANC use. Women who gave birth in 2009 and were sold a voucher booklet had a 3.07 times higher odds of delivering in a health facility.

**Table 4:** Changes in ANC use associated with the intervention

	Unadjusted odds ratios (n = 1,423) (1)	Adjusted odds ratios (n = 1,423) (2)
**Exposure to the voucher scheme**		
No exposure (pre-intervention, 2008)	1.00	1.00
No exposure (post-intervention, 2009)	1.77***	1.74***
Not sold voucher booklet (2009)	1.96	1.93
Sold voucher booklet (2009)	3.07**	4.98***
		
**Program factors unrelated to the voucher scheme**		
Travel time to nearest health facility		
More than five minutes	1.00	1.00
Five minutes or less	2.00***	1.81***
Mass media exposure		
Do not watch television daily	1.00	1.00
Watch television daily	2.06***	1.13
		
**Maternal factors**		
Mother's age		
< 24	1.00	1.00
25-29	1.25*	1.52***
30-34	1.31	1.81**
> 34	0.96	1.63*
Parity/Living children		
1	1.00	1.00
2	0.83	0.73
3	0.66	0.49***
4	0.52***	0.48***
5 or more	0.33***	0.34***
Mother's education		
None	1.00	1.00
Any primary	2.21***	1.73**
Middle	2.22***	1.33
Secondary	5.40***	2.59***
Matriculate or higher	10.18***	3.97***
		
**Household factors**		
Wealth quintiles		
Fifth/poorest	1.00	1.00
Fourth	1.75**	1.31
Third	3.31***	2.24***
Second	5.43***	3.11***
First/least poor	10.33***	4.38***
Mother's autonomy		
Low	1.00	1.00
Medium	1.60**	1.42*
High	1.32	1.12
R-squared		19.17%

*p < 0.05, **p < 0.01, ***p < 0.001

Because women who were targeted for the sale of voucher booklets were different from other women in terms of their demographic and socio-economic characteristics, these differences were adjusted in the regression analysis to determine the effect of the voucher intervention on ANC use.

Column 2 of Table 4 shows adjusted odds ratios associated with a woman making at least three ANC visits during her last pregnancy. After adjusting for other variables, the effect of participation in the voucher scheme became stronger: women who were sold a voucher booklet in 2009 had a five times higher odds of ANC use than women in 2008. The findings also provide evidence of a secular trend in ANC use in D.G. Khan City: the odds of ANC use remained significantly different between women who gave birth in 2009 and were not exposed to the voucher intervention and women who gave birth in 2008 (odds ratio = 1.74).

Travel time to a health facility was associated with ANC use: women living within five minutes of a health facility had a greater likelihood of ANC use (odds ratio = 1.81). After controlling for other factors, daily exposure to the mass media was not associated with ANC use. Women who were ages 25 and older had a higher likelihood of ANC use compared to younger women.

As parity increased, the likelihood of ANC use declined: women with three children had a 0.49 lower odds of ANC use and women with five or more children had a 0.34 lower odds of ANC use. Education increased the likelihood of ANC use: women with any primary education had a 1.73 times higher odds and women with matriculate or higher education had a 3.97 times higher odds of ANC use. Household wealth was associated with higher ANC use: women in the third quintile had a 2.24 times higher odds of ANC use and women in the first quintile had a 4.38 times higher odds of ANC use.

### Effects of voucher scheme on institutional delivery

The impact of the voucher scheme on institutional delivery is shown in Table 5. Column 1 of Table 5 shows unadjusted odds ratios associated with delivery in a health facility. Women who gave birth in 2009 but were not exposed to the voucher intervention were more likely to deliver at a health facility than women who gave birth in 2008 (odds ratio = 1.22). However, this effect did not remain significant once women's demographic and socio-economic characteristics were taken into account (Column 2, Table 5), indicating that there was no secular trend in institutional delivery in D. G. Khan City.

**Table 5:** Changes in institutional delivery associated with the intervention

	Unadjusted odds ratios (n = 1,423) (1)	Adjusted odds ratios (n = 1,423) (2)
**Exposure to the voucher scheme**		
No exposure (pre-intervention, 2008)	1.00	1.00
No exposure (post-intervention, 2009)	1.22*	1.09
Not sold voucher booklet (2009)	1.13	0.98
Sold voucher booklet (2009)	2.58**	4.04***
		
**Program factors unrelated to the voucher scheme**		
Travel time to nearest health facility		
More than five minutes	1.00	1.00
Five minutes or less	1.43**	1.23
Mass media exposure		
Do not watch television daily	1.00	1.00
Watch television daily	1.77***	0.97
		
**Maternal factors**		
Mother's age		
< 24	1.00	1.00
25-29	1.09	1.22
30-34	1.19	1.58*
> 34	0.95	1.64***
Parity/Living children		
1	1.00	1.00
2	0.88	0.79*
3	0.72*	0.58**
4	0.55***	0.54***
5 or more	0.34***	0.34***
Mother's education		
None	1.00	1.00
Any primary	1.84***	1.41***
Middle	1.82**	1.15
Secondary	3.50***	1.79
Matriculate or higher	11.87***	5.03***
		
**Household factors**		
Wealth quintiles		
Fifth/poorest	1.00	1.00
Fourth	1.20	0.94
Third	2.70***	1.88**
Second	3.92***	2.47***
First/least poor	8.00***	3.72***
Mother's autonomy		
Low	1.00	1.00
Medium	1.66*	1.50*
High	1.42*	1.23
R-squared		16.69%

*p < 0.05, **p < 0.01, ***p < 0.001

Women who gave birth in 2009 and were sold a voucher booklet had a 2.58 times higher odds of delivering at a health facility. After adjusting to differences in demographic and socio-economic characteristics (Column 2, Table 5), the effect of participation in the voucher scheme became stronger: women who were sold the voucher booklet had a 4.04 times higher odds of delivering at a health facility.

Parity, education and household wealth had powerful effects on delivery at a health facility. As parity increased, the likelihood of institutional delivery declined: women with two children had a 0.79 times lower odds of institutional delivery and women with five or more children had a 0.34 times lower odds of institutional delivery. Education increased the likelihood of ANC use: women with any primary education had a 1.41 times higher odds of institutional delivery and women with matriculate or higher education had a 5.03 times higher odds of institutional delivery. Wealth was associated with higher odds of institutional delivery: women in the third quintile had a 1.88 times higher odds of institutional delivery and women in the first quintile had a 3.72 times higher odds of institutional delivery.

Programmatic factors that were unrelated to the voucher scheme, including travel time to the nearest health facility and daily exposure to the mass media, were not associated with institutional delivery.

### Effects of voucher scheme on PNC use

The effects of the voucher scheme on PNC use are shown in Table 6. Column 1 of Table 6 shows the unadjusted odds associated with PNC use. Women who gave birth in 2009 and were sold a voucher booklet by outreach workers had a 3.68 times higher odds of making a PNC visit than women who gave birth in 2008. After adjusting for women's demographic and socio-economic characteristics (Column 2, Table 6), this effect became stronger: the odds ratio of PNC use was 5.80 times higher among women who were sold a voucher booklet in 2009 compared to women who gave birth in 2008.

**Table 6:** Changes in PNC use associated with the intervention

	Unadjusted odds ratios (n = 1,423) (1)	Adjusted odds ratios (n = 1,423) (2)
**Exposure to the voucher scheme**		
No exposure (pre-intervention, 2008)	1.00	1.00
No exposure (post-intervention, 2009)	1.30	1.18
Not sold voucher booklet (2009)	1.71	1.53
Sold voucher booklet (2009)	3.68***	5.80***
		
**Program factors unrelated to the voucher scheme**		
Travel time to nearest health facility		
More than five minutes	1.00	1.00
Five minutes or less	1.50**	1.32
Mass media exposure		
Do not watch television daily	1.00	1.00
Watch television daily	1.51*	0.95
		
**Maternal factors**		
Mother's age		
< 24	1.00	1.00
25-29	1.06	1.11
30-34	1.22	1.44
> 34	0.87	1.22
Parity/Living children		
1	1.00	1.00
2	0.79	0.72*
3	0.59***	0.50***
4	0.49**	0.52*
5 or more	0.39***	0.47**
Mother's education		
None	1.00	1.00
Any primary	1.62***	1.33
Middle	2.25***	1.70**
Secondary	3.89***	2.58***
Matriculate or higher	5.58***	3.44***
		
**Household factors**		
Wealth quintiles		
Fifth/poorest	1.00	1.00
Fourth	1.36	1.10
Third	2.70***	2.04***
Second	3.42***	2.21***
First/least poor	5.19***	2.80***
Mother's autonomy		
Low	1.00	1.00
Medium	0.96	0.81
High	1.31	1.13
R-squared		13.62%

*p < 0.05, **p < 0.01, ***p < 0.001

There was no significant difference in the odds of PNC use between women who gave birth in 2009 but were not exposed to the voucher intervention and women who gave birth in 2008. This suggests that there was no secular increase in PNC use between 2008 and 2009.

As parity increased, the likelihood of a woman making a PNC visit declined: women with two children had a 0.72 times lower odds of ANC use and women with five or more children had a 0.47 times lower odds of ANC use. A woman's education and household wealth increased the likelihood of her making an ANC visit: women with middle level education had a 1.70 times higher odds of PNC use and women with matriculate or higher education had a 3.44 times higher odds of PNC use; women from households in the third quintile had a 2.04 times higher odds of PNC use and women from households in the first quintile had a 2.80 times higher odds of PNC use.

### Magnitude of voucher scheme impact

**Figure 4:**
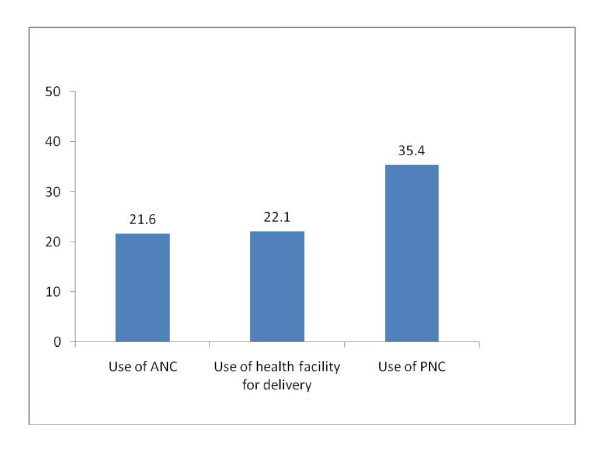
**Logistic Regression estimates of the marginal effect (in percents) of being sold a voucher booklet on the use of maternal health services**.

Figure 4 shows marginal effects estimated from logistic regression analyses that controlled for differences maternal factors, household factors and programmatic factors unrelated to the voucher scheme. These marginal effects were obtained using a post-estimation command in STATA software after running the logistic regression models in Tables 4, 5 and 6.

The estimate of the marginal effect of purchase of a voucher booklet on ANC use was 21.6%. This means that a 21.6 percentage point difference in PNC use was found between women who gave birth in 2009 and were sold a voucher booklet and women who gave birth in 2008. The marginal effect of purchase of a voucher booklet on institutional delivery was 22.1%. In other words, the difference in institutional delivery between women sold a voucher booklet in 2009 and women in 2008 was 22.1 percentage points. The impact of the intervention on PNC use was even larger: there was a 35.4 percentage point difference in PNC use between women who gave birth in 2009 and were sold vouchers and women who gave birth in 2008.

## Discussion

PBF is a relatively new approach being adopted in developing countries to lower maternal mortality by increasing the use of maternal health services, particularly delivery at a health facility. A voucher scheme implemented through medical providers in Gujarat state, India, may have contributed to a large increase in institutional deliveries: over a 14-month period, the Gujarat scheme covered 160,000 deliveries and coverage of deliveries increased by 21 percentage points during the period that the voucher intervention was implemented [20]. However, there remains a lack of convincing evidence regarding the impact of PBF schemes on changes in maternal health services utilization at the population level. This study adds to the evidence base on the effects of demand-side financing interventions to increase the use of health facilities for deliveries and other maternal health services.

The maternal health voucher intervention assessed in this study was intended to cover 2,000 deliveries, and the use of ANC and PNC associated with those deliveries. The findings of the assessment show that participation in the voucher scheme was associated with a 21.6 percentage point increase in institutional delivery in D.G. Khan City, after adjusting for demographic and socio-economic factors. The substantial increase in institutional delivery associated with the voucher intervention becomes even more important given that there was no secular trend showing an increase in institutional delivery during the study period. In other words, in the absence of the voucher scheme, there may have been no increase in institutional delivery in D.G. Khan City. This is consistent with the extremely slow overall change observed when a comparison of the rate of institutional delivery in D.G. Khan City in 2008 (at 61%) is made with the rate of institutional delivery in urban D. G. Khan district in 2005 (at 55%) [16].

The study showed that the very substantial differential in the institutional delivery rate between the poorest and the least poor women in D. G. Khan City (32% vs. 85%) in 2008 was substantially reduced (53% vs. 87%) within the course of a one-year maternal health voucher project, by 2009. Voucher recipients had similar levels of physical access to health services, and similar levels of access to mass media compared to women who were contacted by outreach workers but not sold vouchers and to women who were not contacted by outreach workers. The primary differences between women who were sold voucher booklets and other women were in demographic and socio-economic characteristics. The primary mechanism through which the voucher intervention appears to have contributed to the substantial increase in institutional delivery among voucher recipients was by enhancing their ability to pay for institutional delivery. Yet, the role of outreach workers cannot be underestimated. Multiple visits were made by outreach workers before voucher booklets were purchased by low-income women: the $1.25 price of the booklet was not insubstantial to this segment of the population. These visits were important in convincing women that delivery in a health facility was safer than delivery at home and were critical in overcoming barriers such as opposition by the woman's husband or mother-in-law to delivery in a health facility.

Possibly because of its low use prior to the intervention, the voucher scheme's effect on PNC was particularly dramatic: participation in the voucher scheme was associated with a 35 percentage point increase in PNC. No secular trend was observed for PNC use either. In other words, independent of the effect of other variables, there was no increase in PNC use. Use of PNC remains low in Pakistan overall [8].

The only maternal health service for which a secular trend was observed was ANC. Findings from other studies also suggest that ANC use has been increasing in urban Pakistan in recent years.

Programmatic variables unrelated to the voucher intervention such as travel time to the nearest health facility or television viewership did not have significant effects on institutional delivery or PNC use. However, being within five minutes of the nearest health facility was associated with higher use of ANC. Among non-program variables, parity and mother's education had consistently large effects on the use of maternal health services.

The total cost of implementing and monitoring the D. G. Khan voucher scheme, including the cost of vouchers and staff salaries, was $143,810. The cost of the research study was $24,600. A crude discussion of the cost-effectiveness of the project is possible. No maternal death was observed among women who received voucher booklets. With the Maternal Mortality Ratio in Pakistan at 276 per 100,000 live births [8], the number of maternal deaths prevented by the project can be estimated: our rough calculations suggest that 5.5 maternal deaths may have been prevented by the project, at a cost per life saved of $26,053.

A rough calculation of the cost of eliminating maternal mortality in Pakistan using this PBF approach is also possible. About 13,000 maternal deaths occur in Pakistan annually. If the D. G. Khan voucher scheme were to be translated into a national program, the total cost of eliminating maternal mortality in Pakistan would be about $339 million. While this may appear to be expensive, two factors should be kept in mind: economies of scale may be expected when this type of voucher scheme is implemented at the national level; well-funded maternal health projects in Pakistan have failed to show any positive impact on the institutional deliver rate among the poorest women [9]. Hence, a highly targeted PBF approach such as this one is likely to be considerably more cost-effective than the available alternatives.

This study was based on a pre-test/post-test design. The lack of a control area with which to compare the findings of the project is a major limitation of the study. The wide range of variables controlled for in the regression analysis should diminish or eliminate the effects of selectivity on observed variables. However, the effect of unobserved variables cannot be ruled out. Quasi-experimental pre-test/post-test designs with control areas are stronger designs that should be adopted for the evaluation of large-scale voucher interventions. Two studies with quasi-experimental designs to estimate the effects of maternal health voucher interventions using a strengthened version of the D.G. Khan voucher project are currently being implemented in two districts of Pakistan: Charsadda and Jhang [17]. The findings from those studies should help collect stronger evidence regarding the impact of voucher interventions in increasing the rate of institutional delivery as well as the use of other maternal health services in Pakistan.

## Conclusions

Unless interventions to substantially increase institutional delivery rates are implemented in Pakistan in the very near future, Pakistan will be unable to meet the MDG target of greater than 90% skilled birth attendance by 2015. Unfortunately, there is little evidence in Pakistan of interventions that have succeeded in increasing rates of institutional delivery. Maternal health vouchers appear to be a powerful tool for rapidly increasing institutional delivery rates among the Pakistani poor. While findings from studies with more robust designs are awaited, our conclusions support a rapid and substantial scale-up of interventions using this approach.

## Competing interests

The author is an employee of Population Services International and serves as a technical advisor to Greenstar Social Marketing.

## Authors' contributions

SA was responsible for the design of the study, the data analysis and for the write-up of the report.
